# Five-Year Outcome of Aflibercept Monotherapy for Exudative Age-Related Macular Degeneration with Good Baseline Visual Acuity

**DOI:** 10.3390/jcm10051098

**Published:** 2021-03-05

**Authors:** Wataru Kikushima, Yoichi Sakurada, Atsushi Sugiyama, Seigo Yoneyama, Mio Matsubara, Yoshiko Fukuda, Kenji Kashiwagi

**Affiliations:** Department of Ophthalmology, University of Yamanashi, Chuo Yamanashi 409-3898, Japan; wkikushima@yamanashi.ac.jp (W.K.); asugiyama@yamanashi.ac.jp (A.S.); syoneyama@yamanashi.ac.jp (S.Y.); miom@yamanashi.ac.jp (M.M.); ysugiyama@yamanashi.ac.jp (Y.F.); kenjik@yamanashi.ac.jp (K.K.)

**Keywords:** aflibercept monotherapy, polypoidal choroidal vasculopathy, neovascular age-related macular degeneration, good baseline visual acuity

## Abstract

We investigated the long-term visual and anatomical outcomes of aflibercept monotherapy for exudative age-related macular degeneration (AMD) with good baseline best-corrected visual acuity (BCVA). A medical chart review was performed for 40 consecutive patients with baseline decimal BCVA ≥ 0.6 secondary to exudative AMD. Three monthly injections were administrated, and thereafter additional injection was performed if needed over 5 years. In total, 13 eyes with neovascular AMD (nAMD) and 27 eyes with polypoidal choroidal vasculopathy (PCV) were enrolled. In both groups, the mean BCVA significantly improved at the 12-month visit (*p* < 0.05). However, the significant improvement in BCVA disappeared at the 24-month visit, and the final mean BCVA was equivalent to that at baseline (*p* = 0.17 in the nAMD group and *p* = 0.15 in the PCV group). The median number of injections required after the loading dose was 15.0 during the 5-year follow-up (nAMD:15.0 vs. PCV:15). During the study period, 37 (92.5%) eyes required retreatment(s). Cox regression analysis demonstrated that the protective allele of *ARMS2* A69S was associated with a retreatment-free period from the initial injection (*p* = 0.041, repeated forward selection method). As-needed aflibercept monotherapy is a preferable treatment option for exudative AMD with good initial visual acuity regardless of nAMD or PCV during the 5-year study period.

## 1. Introduction

Age-related macular degeneration (AMD), one of the leading causes of legal blindness in advanced countries with older-aged populations [1], is a chronic inflammatory disease with a varied etiology [2]. The intravitreal injection of vascular endothelial growth factor (VEGF) inhibitors has greatly changed the treatment of exudative AMD. It was first reported that a monthly administration of ranibizumab improved the best corrected visual acuity (BCVA) in eyes with exudative AMD in the ANCHOR/MARINA [3,4]. However, a subsequent study, PIER, demonstrated that the quarterly administration of ranibizumab after three-monthly loading injections failed to improve BCVA in eyes with exudative AMD [5]. On the other hand, the PRONTO study demonstrated that monthly monitoring and as-needed reinjection after three monthly loading ranibizumab injections is an effective treatment option for improving BCVA [6]. Currently, anti-VEGF therapy has become the first-line treatment for exudative AMD worldwide; however, repeated injections after the loading phase are required for most eyes [7]. Therefore, the management of recurrence is essential for patients with AMD to maintain good vision for their lifetime.

Aflibercept is a fusion protein that has a stronger binding affinity to VEGF-A in comparison with ranibizumab and bevacizumab. The VIEW1/2 study demonstrated that bimonthly aflibercept monotherapy after three monthly loading injections is comparable to monthly ranibizumab monotherapy for visual improvement [8]. Many studies have reported favorable outcomes of intravitreal aflibercept injections (IAI) for exudative AMD with various treatment protocols, including fixed interval dosing, as-needed regimen, and treat-and-extend (TAE) regimens [8,9,10,11]. However, the study period was ≤ 24 months in most studies. In large-scale randomized studies, the inclusion baseline best-corrected visual acuity (BCVA) is ≤ 20/40 [3,4]; therefore, there have been few reports investigating the long-term visual outcome in patients secondary to exudative AMD with good baseline visual acuity.

In the present study, we investigated the 5-year visual and anatomic outcomes for patients secondary to exudative AMD with good BCVA ≥ 0.6, who were initially administrated three monthly aflibercept monotherapy followed by as-needed injection.

## 2. Methods

### 2.1. Participants

A retrospective medical chart review was performed in consecutive treatment-naïve eyes secondary to neovascular AMD (nAMD) or polypoidal choroidal vasculopathy (PCV) with baseline BCVA ≥ 0.6 in the decimal scale, receiving 3 monthly intravitreal aflibercept injections (IAIs) at the University of Yamanashi Hospital between January 2013 and July 2015. The present study was approved by the Institutional Review Board of the University of Yamanashi and was conducted as per the tenets of the Declaration of Helsinki. Written informed consent was obtained from all patients.

At baseline, all patients underwent comprehensive ophthalmic examinations, including the measurement of BCVA using Landolt chart, intraocular pressure, slit-lamp biomicroscopy with +78-diopter (D) lens, color fundus photography, fluorescein angiography (FA), indocyanine green angiography (ICGA) using a confocal laser scanning system (HRA-2; Heidelberg Engineering, Dossenheim, Germany), and spectral domain optical coherence tomography (SD-OCT) examination (Spectralis version 5.4 HRA + OCT).

We included patients secondary to exudative AMD, including PCV and nAMD. Eyes with nAMD show type 1 or type 2 neovascularization on SD-OCT and the absence of polypoidal lesions on ICGA. PCV shows a solitary or cluster of polypoidal lesions with or without branching vascular networks on ICGA [12]. Lesion size was defined as the greatest linear dimension (GLD). The GLD, which was defined as the fundus lesion covering the dye leak, pigment epithelial detachment, subretinal hemorrhage, and choroidal neovascularization, if present, was determined using the FA image. The central retinal thickness (CRT) was defined as the vertical distance between the inner border of the retinal pigment epithelium and the inner limiting membrane at the center of the macula in the SD-OCT image. The subfoveal choroidal thickness (SCT) was measured as the vertical distance between the outer border of the retinal pigment epithelium and the chorioscleral border, using the enhanced depth imaging mode equipped with HRA2 Spectralis ver 5.4.

The inclusion criteria were as follows: (1) eyes with treatment-naïve exudative AMD, including nAMD and PCV; (2) baseline decimal BCVA ≥ 0.6 in the Landolt chart; (3) receiving 3 monthly IAI (2.0 mg/0.05 mL); and (4) a follow-up period of 60 months after the initial IAI. The exclusion criteria were (1) previous treatment history for exudative AMD, including intravitreal injection of ranibizumab or photodynamic therapy; (2) eyes that had undergone vitrectomy; (3) eyes with retinal angiomatous proliferation; and (4) other macular abnormalities including myopic choroidal neovascularization (CNV), angioid streaks, and other secondary CNV. If both eyes were eligible, the second eye was included in this study.

### 2.2. Follow Up

All patients received IAIs (2.0 mg/0.05 mL) every 3 months, followed by monthly follow-up requiring SD-OCT examination, as well as routine ophthalmic examination. If residual or recurrent exudation was observed on SD-OCT, retreatment with a single IAI was performed. All patients underwent monthly follow-up. When the eyes had not experienced lesion reactivation for more than 12 months, the follow-up interval was extended for a maximum of 2 months until recurrent exudation was observed. If massive subfoveal hemorrhage, dense vitreous hemorrhage, or cataract progression was observed, appropriate surgical procedures, including pars plana vitrectomy and cataract surgery, were performed. The patients who did not complete the 5-year follow-up period were excluded from this study.

### 2.3. Genotyping

We genotyped two major single nucleotide polymorphisms associated with AMD, namely rs800292 in the *CFH* gene, and rs10490924 in the *ARMS2* gene. Genomic DNA was purified using a PUREGENE DNA Isolation Kit (Gentra Systems, Minneapolis, MN, USA) from the peripheral blood of the participants collected at the time of baseline FA/ICGA. Genotyping of the two genes was conducted using TaqMan genotyping assays with a 7300/7500 real-time polymerase chain reaction system (Applied Biosystems, Foster City, CA, USA) following the manufacturer’s recommendations as previously described [13]. In detail, TaqMan genotyping assays contain sequence-specific primers to amplify the polymorphic sequence of the target genes, and two minor groove binders to stabilize the samples. Purified wet genomic DNA was mixed with a TaqMan genotyping assay and dispensed onto a reaction plate, and the genotyping with a real-time PCR system was performed. The allelic discriminatin plot was collected and analyzed by three researchers (W.K., Y.S., and S.Y.), and recorded on an anonymous basis.

### 2.4. Statistical Analysis

Statistical analyses were performed using the SPSS for Windows (SPSS, Tokyo, Japan). BCVA measured in the decimal scale with a Landolt chart was converted into a logarithm of the minimal angle resolution (log MAR). The differences between continuous and categorical variables were tested using the Mann–Whitney U test, the Kruskal–Wallis test, or the chi-square test. The paired *t*-test was used to determine the significance of the difference between values before and after treatments. Multivariate logistic regression analysis was performed to investigate the baseline risk factors for retreatment due to residual or recurrent exudation. Cox regression survival analysis was conducted to estimate the relative risk for retreatment. *p*-values less than 0.05 were considered statistically significant.

## 3. Results

### 3.1. Change in Log MAR BCVA

A total of 40 eyes of 40 patients were included in the study. The mean age was 71.8 ± 8.0, and 31 patients were male (77.5%). Table 1 shows the baseline characteristics of the patients. There were 13 patients with nAMD (nAMD group) and 27 with PCV (PCV group). There was no significant difference between the two groups except for the baseline BCVAand the risk allele frequency of *ARMS2* and *CFH*. The patients in the PCV group had better baseline BCVA (Mann–Whitney U test) and lower risk allele frequency for *ARMS2* A69S (rs10490924) and *CFH* I62V (rs800292, Chi-square test, Table 1). However, after adjustment for multiple testing by applying the Benjamini Hochberg method, these three variables were also insignificant.

**Figure 1:**
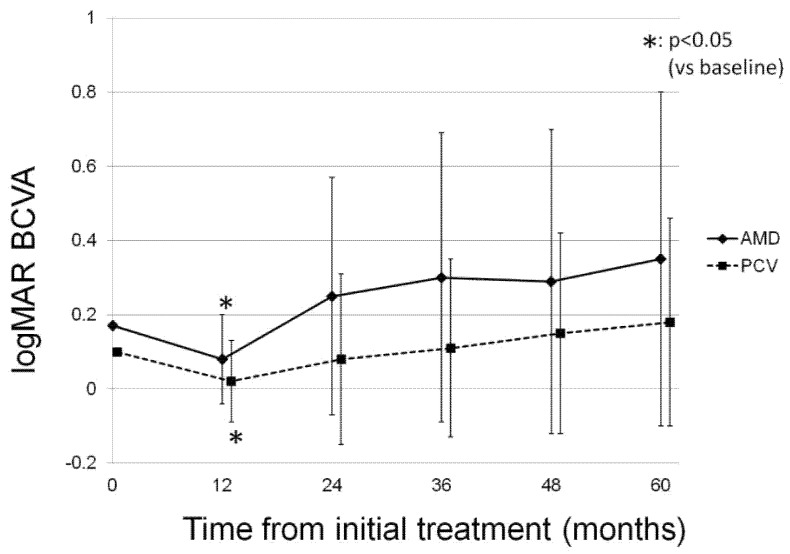
The change in mean best-corrected visual acuity of the participants in each group during the follow-up period. In the neovascular age-related macular degeneration group, the mean BCVA was 0.17 ± 0.07 at baseline, and 0.08 ± 0.12, 0.25 ± 0.32, 0.30 ± 0.39, 0.29 ± 0.41, and 0.35 ± 0.45 at 12 months, 24 months, 36 months, 48 months, and 60 months, respectively (*p* = 0.03, 0.39, 0.25, 0.31, 0.17, respectively). In the polypoidal choroidal vasculopathy group, the mean BCVA was 0.10 ± 0.09 at baseline, and 0.02 ± 0.11, 0.08 ± 0.23, 0.11 ± 0.24, 0.15 ± 0.27, and 0.18 ± 0.28 at 12 months, 24 months, 36 months, 48 months, and 60 months, respectively (*p* = 1.5 × 10^−3^, 0.82, 0.80, 0.24, 0.15, respectively).

The change in mean BCVA of the patients in each group during the follow-up period is presented in Figure 1. Although the mean BCVA in each group significantly improved at 12 months from the baseline (*p* < 0.05), the significance disappeared at 24 months, and the final mean BCVA was equivalent to that at baseline (*p* = 0.17 in the nAMD group and *p* = 0.15 in the PCV group). To evaluate the reason for the decrease in BCVA improvement after month 12, we reviewed the SD-OCT images of the patients with a final BCVA deterioration of 0.3 logMAR or worse. The result revealed that there were 7(17.5%) patients with severe BCVA deterioration due to various macular or other ocular pathologies, including macular atrophy in two eyes, not achieving dry macula in two eyes, subfoveal hemorrhage from PCV in one eye, cataract in one eye, and macular edema secondary to central retinal vein occlusion in one eye.

**Figure 2:**
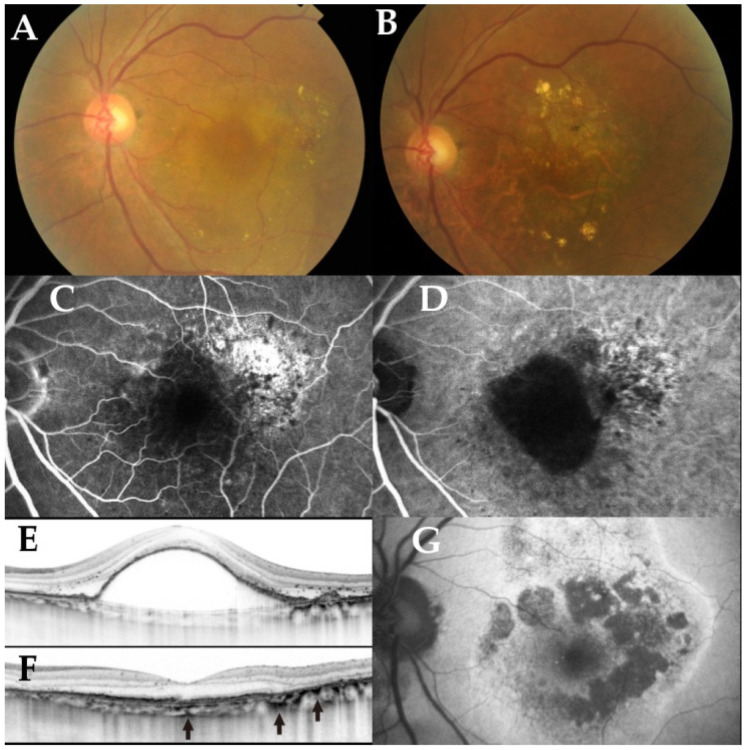
A representative case of a 69-year-old female with type 1 neovascularization in the left eye. (**A**) Color fundus photography in the left eye at baseline reveals serous retinal detachment (SRD) and pigmental epithelial detachment (PED) in the macula. (**B**) Color fundus photography in the left eye at 60 months from baseline reveals absorption of SRD and retinal pigment epithelium (RPE) atrophy around the fovea. (**C**) Fluorescein angiography (FA) of the left eye at baseline demonstrates window defect at the temporal of the fovea. (**D**) Indocyanine green angiography demonstrates PED including fovea as a hypofluorescent lesion. (**E**) Spectral domain optical coherence tomography (SD-OCT) of fovea in the left eye at baseline reveals large PED with double-layer sign and SRD. (**F**) SD-OCT of fovea in the left eye at 60 months from baseline reveals complete resolution of PED and SRD; however, choroidal hypertransmission due to subfoveal RPE atrophy is apparent (black arrows). (**G**) Fundus autofluorescense of the left eye at 60 months from baseline reveals RPE atrophy as hypoautofluorescent areas around the fovea.

Figure 2 presents a representative case with severe vision. The patient required 32 IAIs after initial three monthly injections during a 5-year follow-up period. Though her initial BCVA was 0.8 on the decimal scale, macular atrophy had developed since month 48, resulting in a deteriorated BCVA of 0.1 at month 60.

To investigate the factors associated with BCVA change at 60 months, we conducted a multivariate regression analysis as a multiple comparison (Table 2). The results showed that female sex was associated with good BCVA improvement at 60 months. During the follow-up period, four patients underwent cataract surgery. However, the history of cataract surgery was not associated with BCVA improvement at 60 months (Table 2). None of the eyes required vitrectomy.

### 3.2. Change in OCT Parameters

**Figure 3:**
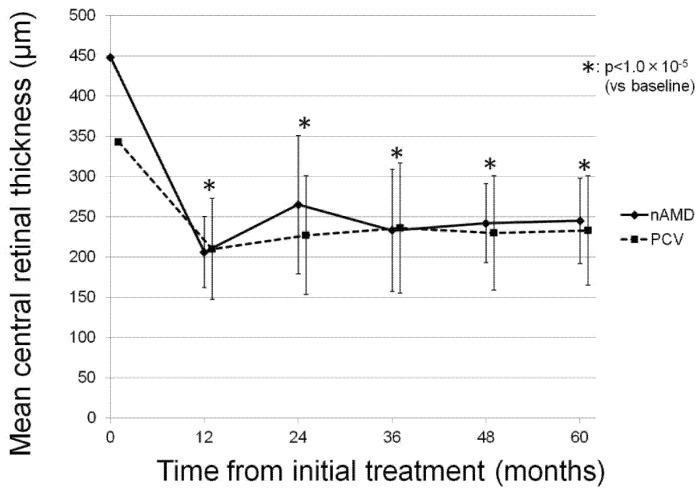
The change in mean central retinal thickness in each group during the follow-up period. Mean central retinal thickness (CRT) shows significant decreases from 448 ± 231 µm at baseline to 206 ± 44 µm at 12 months, 265 ± 86 µm at 24 months, 233 ± 76 µm at 36 months, 242 ± 49 µm at 48 months, and 245 ± 53 µm at 60 months (*p* = 2.5 × 10^−3^, 9.1 × 10^−3^, 2.4 × 10^−3^, 6.9 × 10^−3^, 3.1×10^−3^, respectively) in the neovascular age-related macular degeneration group, and mean CRT shows a significant decrease from 343 ± 88 µm at baseline to 210 ± 63 µm at 12 months, 227 ± 74 µm at 24 months, 236 ± 81 µm at 36 months, 230 ± 71 µm at 48 months, and 233 ± 68 µm at 60 months (*p* = 1.0 × 10^−5^, 1.0 × 10^−5^, 4.0 × 10^−4^, 1.0 × 10^−5^, 1.0 × 10^−5^, respectively) in the polypoidal choroidal vasculopathy group.

During the follow-up period, the mean CRT of all participants significantly decreased from 376.7 ± 155.1 µm at baseline to 237.1 ± 62.7 µm at 60 months (*p* < 0.05). A significant reduction in CRT was also observed in both the nAMD and PCV groups (*p* < 0.05). Figure 3 illustrates the change in mean CRT in each group.

**Figure 4:**
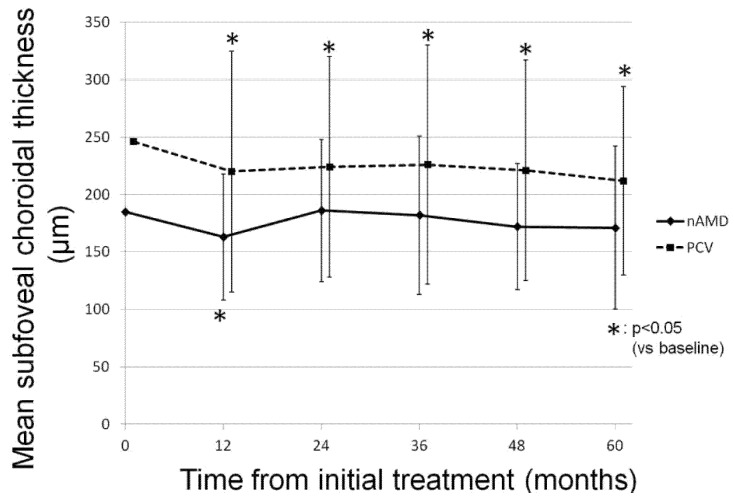
The change in mean subfoveal choroidal thickness in each group during the follow-up period. In the neovascular age-related macular degeneration group, mean subfoveal choroidal thickness (SCT) was 185 ± 56 µm at baseline, and 163 ± 55 µm, 186 ± 62 µm, 182 ± 69 µm, 172 ± 55 µm, and 171 ± 71 µm at 12 months, 24 months, 36 months, 48 months, and 60 months, respectively (*p* = 0.013, 0.95, 0.82, 0.28, and 0.37, respectively). In the polypoidal choroidal vasculopathy group, mean SCT showed a significant decrease from 246 ± 108 µm at baseline to 220 ± 105 µm at 12 months, 224 ± 96 µm at 24 months, 226 ± 104 µm at 36 months, 221 ± 96 µm at 48 months, and 212 ± 82 µm at 60 months (*p* = 6.0 × 10^−5^, 2.9 × 10^−4^, 3.3 × 10^−3^, 1.8 × 10^−3^, 1.9 × 10^−3^, respectively).

Similar to CRT change, the mean SCT of all participants significantly decreased from 226.0 ± 97.8 µm at baseline to 198.8 ± 80.0 µm at 60 months (*p* < 0.05). In the nAMD group, the mean SCT significantly decreased at 12 months from baseline (*p* < 0.05); however, the final mean SCT reduction was not significant at 60 months (*p* = 0.37). In contrast, in the PCV group, a significant decrease in mean SCT was maintained throughout the follow-up period (*p* < 0.05). Figure 4 shows the changes in the mean SCT in each group.

### 3.3. Retreatment

**Figure 5:**
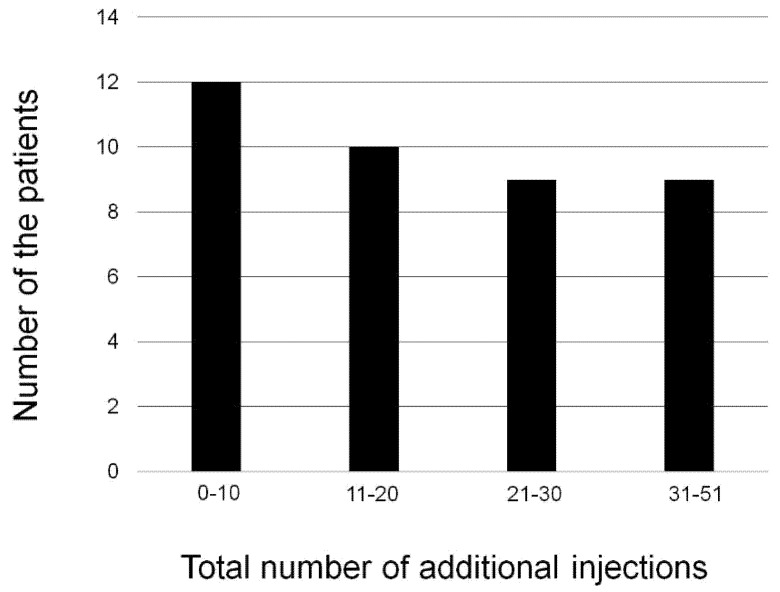
The distribution of patients according to the number of total injections required after the loading dose. In total, 12 (30%) patients were administered 0–10 injections, 10 (25%) patients 11–20 injections, 9 (22.5%) patients 21–30 injections, and 9 (22.5%) patients were administered 35–51 injections during the follow-up period. The median and mean numbers of intravitreal aflibercept injections required after the loading dose in all patients are 15.0 and 18.4 ± 14.0.

Among the 40 eyes, 37 (92.5%) needed IAI(s) as retreatment after the loading dose during the 60-month follow-up period. Figure 5 shows the distribution of patients according to the number of total injections required after the loading dose. Table 3 shows the mean number of total injections required after the loading dose and the distribution of mean number of injections year-by-year. There is no significant difference in the mean number of IAIs between the two groups (21.2 ± 14.2 in the neovascular AMD group and 17.1 ± 13.9 in the PCV group, *p* = 0.36). We conducted a multivariate linear regression analysis to investigate the factors associated with the number of total injections required after the loading dose as a multiple comparison. As shown in Table 4, the risk allele of *ARMS2* A69S (rs10490924) was the only factor associated with the number of total injections.

**Figure 6:**
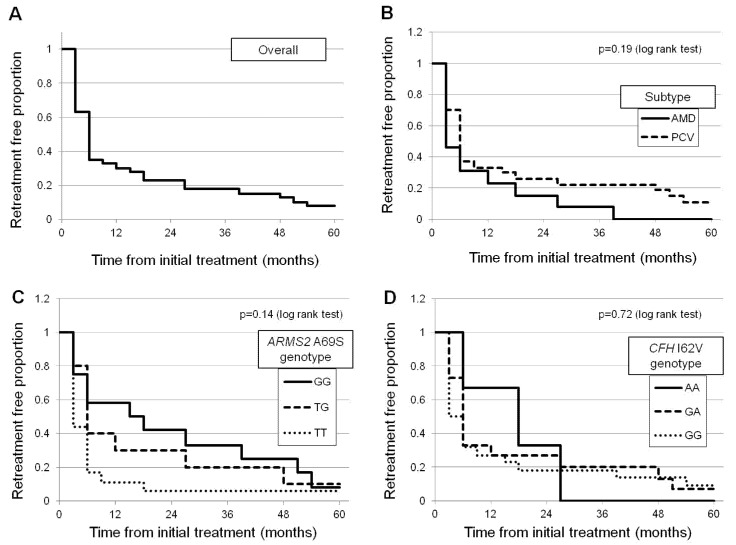
Kaplan–Meier curve regarding retreatment-free proportion from the initial injection. (**A**) The mean retreatment-free period after the initial intravitreal aflibercept injections (IAI) is 16.1 ± 18.7 months, and the retreatment-free proportion at 60 months is 7.5% in all patients. (**B**) The mean retreatment-free period is 11.2 ± 11.5 months and 18.5 ± 21.1 months in the nAMD and PCV groups, respectively. The retreatment-free proportion at 60 months is 0% and 11% in the nAMD group and the PCV group, respectively (*p* = 0.19, log-rank test). (**C**) Mean retreatment-free period is 24.6 ± 18.8 months, 18.8 ± 20.1 months, and 9.0 ± 13.2 months in patients with GG, TG, and TT genotypes of *ARMS2* A69S, respectively. The retreatment-free proportion at 60 months is 8%, 10%, and 6% in patients with GG, TG, and TT genotypes of *ARMS2* A69S, respectively (*p* = 0.14, log-rank test). (**D**) The mean retreatment-free period is 17.7 ± 10.7 months, 17.4 ± 19.6 months, and 15.0 ± 19.5 months in patients with AA, AG, and GG genotype of **CFH** I62V, respectively. The retreatment-free proportion at 60 months is 0%, 7%, and 9% in patients with AA, AG, and GG genotypes of **CFH** I62V, respectively (*p* = 0.72, log-rank test).

To investigate the factors associated with retreatment, a Cox regression survival analysis was conducted. The results showed that the protective allele (G allele) of *ARMS2* A69S (rs10490924) was the only factor associated with a retreatment-free period from the initial injection (β-coefficient = 0.42, *p* = 0.041, repeated forward selection method). Other variables including age, sex, baseline BCVA, baseline CRT, baseline SCT, or allele of *CFH* I62V (rs800292) were not associated with a retreatment-free period. Figure 6 shows the Kaplan–Meier curve for a retreatment-free proportion from the initial injection depending on disease subtypes, *ARMS2* A69S, and *CFH* I62V genotypes.

## 4. Discussion

To the best of our knowledge, this is the first study investigating the long-term treatment outcome of as-needed IAIs for exudative AMD in patients with relatively good initial visual acuity. Our study was a retrospective cohort study; therefore, we consider it as valuable as real-world data.

In the literature, there have been several studies evaluating the long-term efficacy and safety of anti-VEGF agents, including aflibercept, for exudative AMD. Khanani et al. reported on the five-year outcome of an intravitreal anti-VEGF agent treatment using a TAE regimen for nAMD in eyes with baseline vision 20/60 or better. They described that more than 75% of the patients could maintain their baseline VA, and the mean number of injections during years 1–5 was 7.9, 5.9, 5.6, 5.9, and 6.0, respectively [14]. Javidi et al. reported the treatment outcome of a similar intravitreal drug therapy for nAMD using a TAE regimen for up to 7 years [15]. The results were also preferable; the mean BCVA was maintained with a mean number of injections of 7.6 during the first three years, and 5.9 during years 4–7. The difference between our study and these studies is that we selected IAI only for the intravitreal treatment with an as-needed regimen, whereas they had chosen not only aflibercept but also ranibizumab or bevacizumab using the TAE regimen. As a result, the patients in our study achieved similar visual outcomes with relatively fewer additional injections than those in their studies.

Maguire et al. reported the long-term treatment outcomes of anti-VEGF agents, including bevacizumab and ranibizumab, for treating nAMD in an extended comparison of AMD treatments trial study [16]. They reported that the mean change in visual acuity at 5 years from baseline was −3 letters. In the present study, the mean BCVA changed from 0.12 ± 0.09 at baseline to 0.23 ± 0.35 at 60 months, and the BCVA change was equivalent to −6 letters on the early treatment diabetic retinopathy study chart. Given that the initial visual acuity in the present study was equal to or better than the decimal BCVA of 0.6, we consider that the as-needed aflibercept therapy was an effective treatment option for exudative AMD, even in patients with good initial BCVA. However, in the present study, a significant improvement in mean BCVA was apparent at 12 months in both the nAMD and PCV groups; thereafter, the mean BCVA returned to the baseline values and was maintained throughout the follow-up period. We consider that the discrepancy between the insignificant BCVA improvement and the significant reduction in CRT or SCT at month 60 was due to the patients with macular or other pathologies, which were independent of CRT of SCT. In the present study, 7 of 40 patients showed BCVA deterioration of 0.3 logMAR or worse due to these conditions. Even if these patients with severe BCVA deterioration were excluded from the analysis, it did not reach a statistical significance between baseline BCVA and BCVA at 60 months.

During a 60-month follow-up period, 37 (92.5%) out of 40 eyes needed a minimum of one retreatment. Cox regression analysis revealed that the protective allele of *ARMS2* A69S was associated with a retreatment-free period from the initial injection. In a previous study, we investigated the short-term prognostic factors associated with retreatment among patients who received an initial 3-month IAI for exudative AMD [17]. Cox regression analysis revealed that the risk allele of the *ARMS2* A69S gene and older age were associated with a shorter retreatment and reactivation time from the initial injection. In the present study, the positive association between the protective allele of *ARMS2* A69S and the retreatment-free period from initial injection was also confirmed even in patients with good baseline BCVA. Recently, we reported that the protective allele of *ARMS2* A69S was associated with a retreatment-free period in patients with PCV who received the combination therapy involving photodynamic therapy (PDT) and intravitreal injection of anti-VEGF agents [18,19]. The differences between the present study and the previous study are the better initial mean BCVA, including the patients with nAMD, and excluding PDT as a treatment modality in the present study. Consequently, there are several differences in the results between the studies. The proportion of the patients who had not required any retreatment was greater in the previous study than in the present study (27.9% vs. 7.5%), and the total number of retreatments during a 5-year follow-up period in the previous study was relatively smaller than that in the present study (7.51 ± 7.25 injections and 0.51 ± 1.01 combination therapies vs. 18.4 ± 14.0 injections). These results might indicate the good potential of PDT in terms of disease stability against PCV. On another front, the preferable results in the present study might shed light on the choice of treatment modality for PCV patients with initial BCVA ≥ 0.8 in the decimal scale, which had not been included in the previous studies. Moreover, from the previous and present results, it might be inferred that the risk allele of *ARMS2* A69S might be associated with lesion reactivation after treatment, regardless of the treatment modality.

There are several limitations to the present study. The first limitation is the retrospective nature of the analysis. The second limitation is the relatively small sample size. The third limitation is the wide fluctuations in the data. The standard deviations of mean BCVA, CRT, and SCT presented in Figure 1, Figure 2 and Figure 3 were high, and the significant P values presented in the tables were slightly below the threshold of *p* < 0.05. These limitations might lead to some uncertainties about the interpretation of the findings. Further prospective analysis of the long-term visual and anatomical outcomes of aflibercept for exudative AMD with a large sample size is necessary.

In conclusion, IAI is a preferable treatment option for exudative AMD for prolonged disease stabilization, even in patients with good initial visual acuity. The risk allele of the *ARMS2* A69S gene is associated with earlier retreatment after the initial three monthly IAIs.

## Figures and Tables

**Table 1:** Baseline characteristics of the subject.

	All Subjects *n* = 40	nAMD *n* = 13	PCV *n* = 27	*p*-Value (nAMD vs. PCV)
Age	71.8 ± 8.0	74.0 ± 9.3	70.8 ± 7.2	0.38
Gender male	31(77.5%)	11(84.6%)	20(74.1%)	0.45
Baseline BCVA	0.12 ± 0.09	0.17 ± 0.07	0.10 ± 0.09	0.02
Baseline CRT	377 ± 155	448 ± 231	343 ± 88	0.28
Baseline SCT	226 ± 98	185 ± 56	246 ± 108	0.10
Baseline GLD	2767 ± 1385	3049 ± 1554	2631 ± 1306	0.41
*ARMS2* A69S (rs10490924)				
GG:TG:TT (T allele frequency)	12:10:18 (57.5%)	1:4:8 (76.9%)	11:6:10 (48.1%)	0.015
*CFH* I62V (rs800292)				
AA:GA:GG (G allele frequency)	3:15:22 (73.8%)	0:3:10 (88.5%)	3:12:12 (66.7%)	0.038

nAMD: neovascular age-related macular degeneration, PCV: polypoidal choroidal vasculopathy.

**Table 2:** Multivariate linear regression analysis of factors associated with mean BCVA change at 60 months.

Variables	β-Coefficient	*p*-Value
Age	0.01	0.25
Male gender	−0.36	0.023
Baseline log MAR BCVA	−0.23	0.76
Greatest linear dimension	−2.8 × 10^−6^	0.95
Central retinal thickness	4.1 × 10^−4^	0.41
Subfoveal choroidal thickness	−4.5 × 10^−4^	0.50
Subtype (nAMD = 0, PCV = 1)	0.02	0.91
*ARMS2* A69S T allele	2.0 × 10^−3^	0.98
*CFH* I62V G allele	0.14	0.16
Cataract surgery	0.046	0.82
Total number of IAIs	5.6 × 10^−4^	0.90

*ARMS2*: age-related maculopathy susceptibility; BCVA: best-corrected visual acuity; *CFH*: complement factor H; IAI: intravitreal aflibercept injection; logMAR: logarithm of the minimal angle resolution.

**Table 3:** Mean number of total injections required after the loading dose and the distribution of the mean number of injections year-by-year.

Total (0–60 Months)	1st Year (0–12 Months)	2nd Year (13–24 Months)	3rd Year (25–36 Months)	4th Year (37–48 Months)	5th Year (49–60 Months)
18.4 ± 14.0	2.4 ± 2.5	3.8 ± 3.1	3.7 ± 3.4	3.8 ± 3.4	4.4 ± 3.9

**Table 4:** Multivariate linear regression analysis of factors associated with number of total injections.

Variables	β-Coefficient	*p*-Value
Age	0.44	0.23
Male gender	−5.61	0.36
Baseline log MAR BCVA	39.3	0.18
Greatest linear dimension	−2.0 × 10^−3^	0.32
Central retinal thickness	0.023	0.22
Subfoveal choroidal thickness	−0.029	0.27
Subtype (nAMD = 0, PCV = 1)	7.09	0.27
*ARMS2* A69S T allele	6.63	0.025
*CFH* I62V G allele	0.87	0.83

*ARMS2*: age-related maculopathy susceptibility; BCVA: best-corrected visual acuity; *CFH*: complement factor H; logMAR: logarithm of the minimal angle resolution.

## Data Availability

We will provide the data if necessary.
